# Optimal Handling of the Patella in Tourniquet‐Free Total Knee Arthroplasty: Eversion or Lateral Retraction?

**DOI:** 10.1111/os.12819

**Published:** 2020-10-28

**Authors:** Mingcheng Yuan, Yichen Wang, Haoyang Wang, Zichuan Ding, Qiang Xiao, Zongke Zhou

**Affiliations:** ^1^ Department of Orthopaedics West China Hospital/West China School of Medicine, Sichuan University Chengdu China

**Keywords:** Patellar eversion, Patellar lateral retraction, Randomized controlled trial, Total knee arthroplasty, Tourniquet

## Abstract

**Objective:**

To explore the optimal handling of the patella during total knee arthroplasty (TKA) without the intraoperative application of a tourniquet.

**Methods:**

A total of 104 patients undergoing primary unilateral TKA without the intraoperative use of tourniquets from December 2018 to March 2019 in our center were included in this prospective randomized double‐blinded study, including 42 men and 62 women with a mean age of 66.3 ± 7.8 years and a minimum follow‐up of 1 year. Patients were randomly divided into an eversion group (*n* = 52) and a retraction group (*n* = 52) based on the intraoperative handling of the patella (eversion or lateral retraction). Primary outcome measures, including the visual analog scale at rest (rVAS) and the visual analog scale in motion (mVAS) for both anterior knee pain and thigh pain, opioid consumption, active range of motion (aROM), passive range of motion (pROM), the time needed for return of the straight‐leg raise (SLR), and 90° knee flexion, were recorded by an independent observer, who also noted secondary outcome measures, including operation time, length of stay (LOS), patella‐related (patellar tilt and baja) and other complications, knee swelling, Hospital for Special Surgery (HSS) scores, and the 12‐item Short Form Health Survey (SF‐12) scores.

**Results:**

There were no significant differences between the two groups in terms of baseline parameters. At 24, 48, and 72 h postoperatively (PO), patients in the eversion group experienced more severe thigh pain than those in the retraction group (24 h: 2.6 ± 0.8 *vs* 2.2 ± 0.5 [*P* = 0.003]; 48 h: 2.0 ± 0.5 *vs* 1.8 ± 0.4 [*P* = 0.026]; 72 h: 1.1 ± 0.4 *vs* 0.9 ± 0.5 [*P* = 0.012], respectively) and consumed more opioids (24 h: 22.3 ± 7.7 *vs* 15.1 ± 8.9 mg [*P* < 0.001]; 48 h: 27.3 ± 9.3 *vs* 21.4 ± 10.5 mg [*P* = 0.003]; 72 h: 23.1 ± 8.2 *vs* 19.8 ± 7.6 mg [*P* = 0.036], respectively), but no significant difference was found in anterior knee pain (both rVAS and mVAS) preoperatively or at 24, 48 or 72 h, 3 weeks, 2 or 6 months, or 1 year PO (*P* > 0.05). Throughout the 1‐year follow‐up, patients in the retraction group showed significantly better function, including greater aROM and pROM at all time points (*P* < 0.05) and a shorter period of time needed for return to SLR (1.9 ± 0.7 *vs* 2.2 ± 0.8 days [*P* = 0.044]) and 90° knee flexion (1.2 ± 0.7 *vs* 1.9 ± 0.8 days [*P* < 0.001]). In addition, no between‐group difference was found in operation time, complication rate, swelling of the knee, or SF‐12 score during the follow‐up period (*P* > 0.05). The eversion group had a significantly longer LOS (5.61 ± 1.92 *vs* 4.93 ± 1.45 days [*P* = 0.044]) and worse HSS score within 2 months PO (3 weeks PO: 77.4 ± 7.6 *vs* 81.7 ± 7.2 [*P* = 0.004]; 2 months PO: 85.1 ± 7.2 *vs* 88.5 ± 6.1 [*P* = 0.011]), but at the third follow‐up (6 months PO), the HSS score in the two groups became comparable (*P* > 0.05). No patellar tilt occurred in either group and there was only one case of patellar baja in the eversion group, with no significant between‐group difference (*P* > 0.05).

**Conclusion:**

We recommend lateral retraction rather than eversion for optimal handling of the patella during TKA because the postoperative functional recovery is better and thigh pain is relatively less severe.

## Graphical abstract

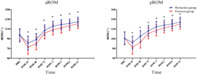

We found significantly better range of motion (ROM), earlier return of straight‐leg raise (SLR), and shorter length of stay (LOS) in the patellar lateral retraction group. Although postoperative anterior knee pain was comparable in the two groups, patients in the patellar eversion group experienced significantly more severe thigh pain and consumed more opioids.

## Introduction

Total knee arthroplasty (TKA) has become one of the most effective treatments for relieving joint pain and improving joint function in patients with end‐stage knee diseases^1^. In TKA, two main techniques are applied to expose the surgical field: patellar lateral retraction and patellar eversion. Debate on the advantages and disadvantages of these two patellar techniques has continued with the increasing popularity of TKA.

In 2004, Bonutti *et al*.^2^ first summarized the techniques for minimally invasive TKA (MITKA), which was reported to provide a series of benefits over conventional TKA, including more rapid functional return, less postoperative anterior knee pain, greater range of motion (ROM) of the knee, less blood loss, and shorter length of stay (LOS)^3^, ^4^, ^5^. The researchers suggested that during MITKA, the patella should be laterally retracted rather than everted to expose the surgical field. The proponents of MITKA attributed the unsatisfactory early rehabilitation and complications such as patella baja and tilt to quadriceps and patellar tendon impairment following intraoperative patellar eversion. They believed that with the patella 180° everted, even without visual damage, the strong tractive force on the quadriceps and patellar tendon would still cause micro scarring, which the postoperative anterior knee pain and shortening of the patella tendon (patella baja) are secondary to, and that patella lateral retraction could perfectly avoid the aforementioned problems^6^, ^7^. However, some others researchers hold the differing opinion that lateral retraction of the patella does not yield superior postoperative results over eversion during TKA^8^, ^9^, ^10^, ^11^. In a random control trial including 66 patients, Reid *et al*.^9^ demonstrated that laterally retracting rather than everting the patella during TKA resulted in no significant benefit in clinical outcomes (pain, Oxford knee score, and other functional evaluation items) or the incidence of patellar tendon shortening postoperatively and in the early to medium follow‐up phase. However, they found that in a lateral retraction group, implant malposition of lateral tibial overhang occurred more frequently, which they blame on the inadequate exposure of the lateral compartment of the knee. Grassi *et al*.^12^, in a systematic review of overlapping meta‐analyses, drew a similar conclusion that although a patella eversion group experienced a relatively shorter tourniquet time intraoperatively, there were no other significant differences in regard to complications, quadriceps strength, and functional and radiological outcomes.

The underlying cause of the controversial conclusions in the research above might be that the surgeons all used tourniquets intraoperatively and that their usage of tourniquets was different (e.g. in time and pressure). In recent decades, an increasing number of researchers have found that the use of tourniquets can cause adverse events, including thigh pain, knee swelling, and other ischemia reperfusion injuries, thereby delaying postoperative recovery; sequelae include less ROM, delayed return of the straight‐leg raise (SLR) and 90° knee flexion, and prolonged LOS^13^, ^14^, ^15^, which overlaps with the outcomes of investigations of patellar eversion or lateral retraction. Both patellar eversion and tourniquet application might cause postoperative thigh pain, less ROM and delayed return of SLR and 90° knee flexion. Therefore, it is difficult to determine exactly what caused the aforementioned adverse events and to compare the difference in outcomes between the two techniques for handling the patella with the tourniquet as a confounder. Currently, surgeons tend to avoid using tourniquets during TKA, but the optimal handling of patellae under tourniquet‐free TKA remains unclear.

Therefore, the aims of this prospective randomized double‐blinded study were: (i) to evaluate the clinical effect of patellar eversion and patellar lateral retraction on postoperative pain and functional recovery after tourniquet‐free TKA; (ii) to compare the postoperative complications following patellar eversion and lateral retraction in tourniquet‐free TKA; and (iii) to make a recommendation on choosing patellar eversion or patellar lateral retraction in TKA.

## Materials and Methods

### *Ethics Statement*

This prospective randomized, controlled, double‐blinded trial was registered in the Chinese Clinical Trial Registry (ChiCTR1900023971). Approval was obtained from the Clinical Trials and Biomedical Ethics Committee of West China Hospital. Written informed consent was obtained from all participants prior to surgery.

### *Inclusion and Exclusion Criteria*

From December 2018 to March 2019, male and female knee osteoarthritis patients of any age who were undergoing primary unilateral tourniquet‐free TKA were eligible for this study. The inclusion criteria were: (i) patients diagnosed with osteoarthritis; (ii) patients treated with primary unilateral tourniquet‐free TKA; (iii) patients who received either patellar eversion or patellar lateral retraction to expose the surgical field intraoperatively; and (iv) preoperative, postoperative, and follow‐up outcomes were evaluated, including primary parameters (visual analog scale at rest [rVAS] and visual analog scale in motion [mVAS] of both anterior knee pain and thigh pain, opioid consumption, active range of motion [aROM], passive range of motion [pROM], the time needed for return of the SLR and 90° knee flexion) and secondary parameters (operation time, LOS, patella‐related [patellar tilt and baja] and other complications, knee swelling, Hospital for Special Surgery [HSS] scores, and the 12‐item Short Form Health Survey [SF‐12] scores).

The exclusion criteria were: (i) body mass index (BMI) of >40; (ii) severe varus or valgus deformity (>20°), and flexion deformity (>40°); (iii) history of knee surgery or knee infection; (iv) anemia (hemoglobin <10 g/dL) or American Society of Anesthesiologists (ASA) grade IV–V; and (v) uncompleted clinical and follow‐up data.

**Fig. 1:**
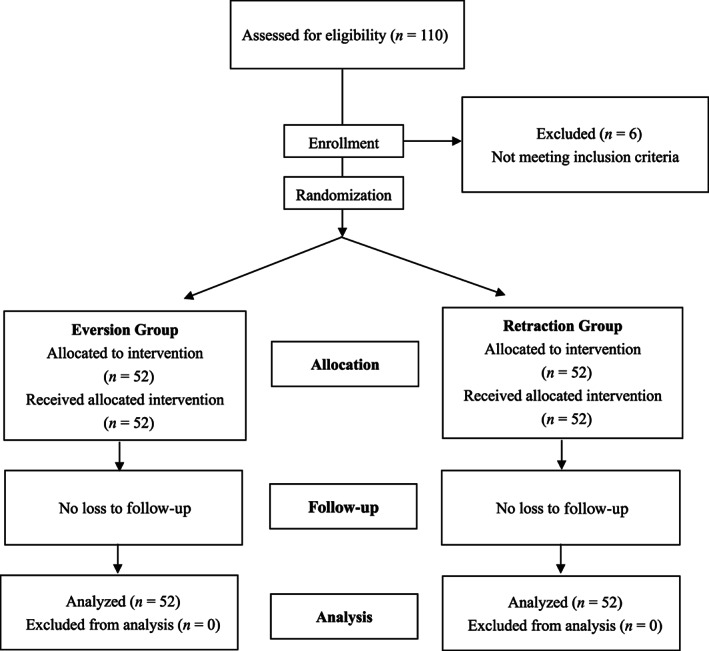
Patient flowchart.

A total of 110 participants were assessed for eligibility. Two were excluded due to a history of knee infection, one for a previous knee surgery, two for being overweight with BMI > 40, and one for severe valgus deformity (35°). No patient was lost to follow‐up, and none of them died during the 1‐year follow‐up period, leaving 52 patients in both the patellar eversion group and the patellar lateral retraction group for the final analysis. The flow of participants through each stage of the study is presented in Fig. 1.

### *Randomization and Blinding*

A computerized random sequence was generated by a nurse who was isolated from the content of the trial to assign the order of randomization. The sequence was concealed *via* a sealed‐envelope method until the interventions were assigned in the operation room. The baseline data and outcome measures were collected by an independent physician, while another independent observer was assigned the task of performing the radiographic evaluation. They were all blinded to the intervention allocation, as were the patients themselves.

### *Surgical Procedure*

#### *Anesthesia and Position*

All TKA were performed by the same team led by a senior admitting orthopaedic surgeon under general anesthesia. The patients were all placed in a supine position. To facilitate the operation procedure, two pedals were placed at the operation side to achieve the 30° and 90° bending of the knee intraoperatively. No tourniquet or drainage tube were applied to any patients.

#### *Approach and Exposure*

**Fig. 2:**
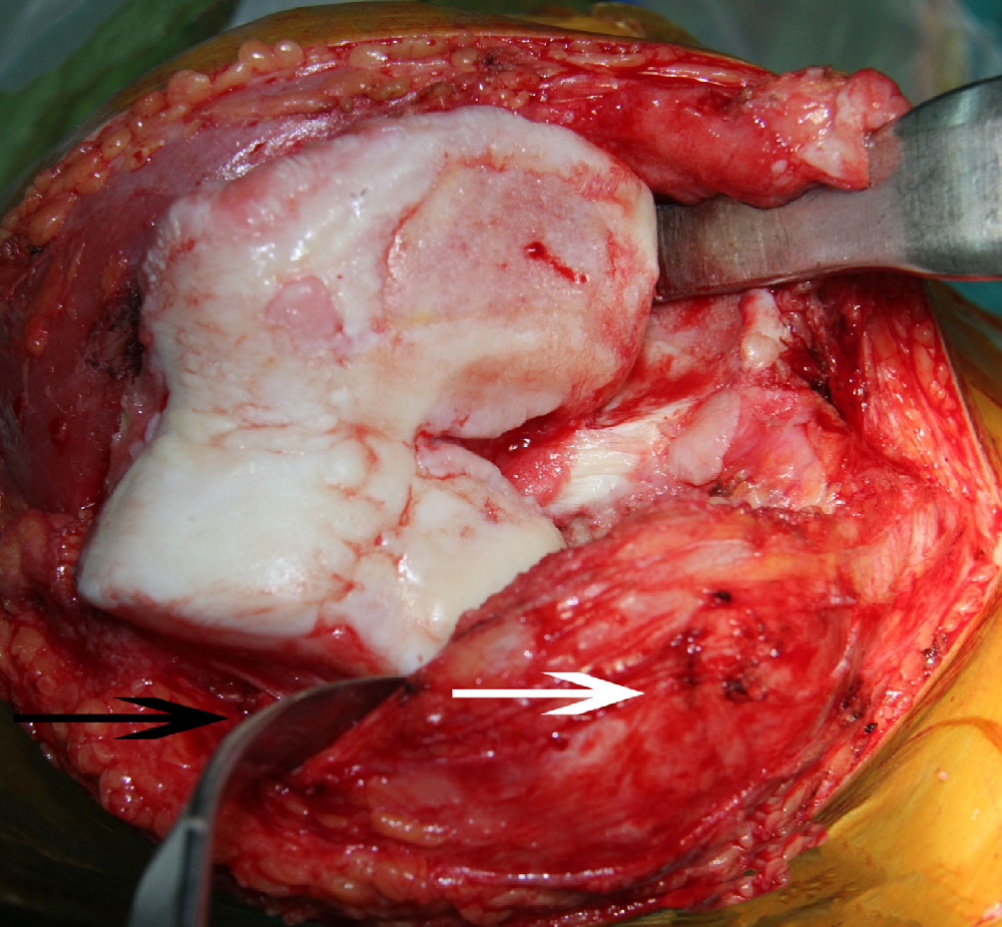
The patella is subluxated outwards by the assistant with a Hoffman retractor. The white arrow is pointing at the patella and the black arrow is pointing at the Hoffman retractor, which subluxated the patella outwards. The pressure exerted on the retractor is relatively small, so the patella is just turned 90° and the tension in both the patellar tendon and the quadriceps tendon are small.

**Fig. 3:**
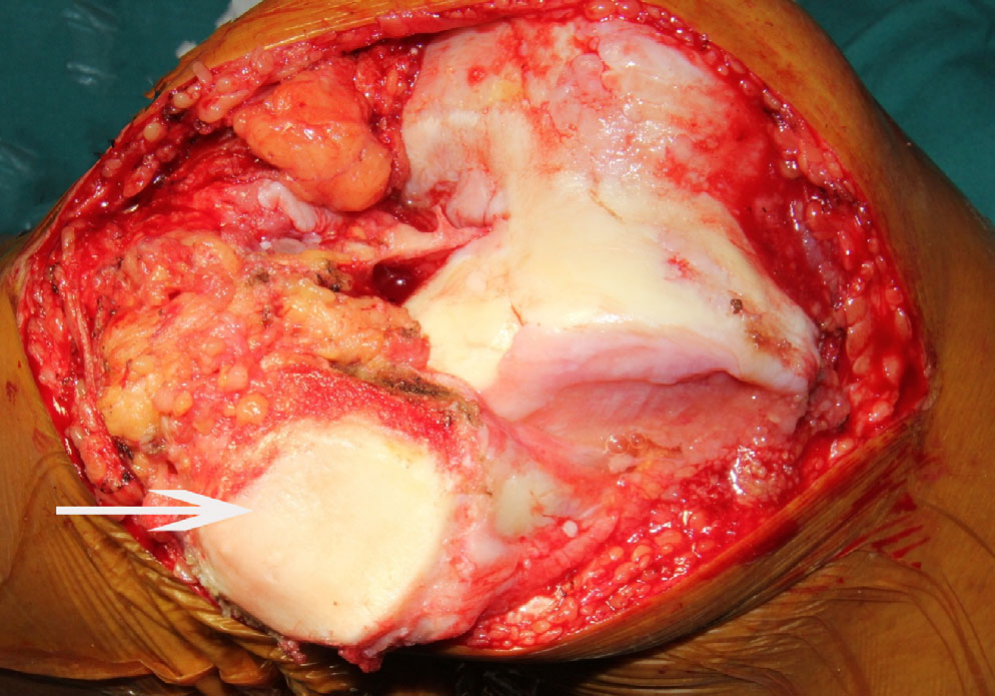
The patella is totally luxated by being rotated 180° with the patellofemoral joint surface upside down. The white arrow is pointing at the patella. The patella is totally turned, so both the patellar tendon and the quadriceps tendon suffer from powerful twist.

A standard TKA procedure was performed through a midline incision and a medial parapatellar approach. The quadriceps tendon was split longitudinally to expose the patella for subsequent eversion or lateral retraction in an extended knee position. In the lateral retraction group, the patella was subluxated outwards by the assistant with a Hoffman retractor (Fig. 2). In the eversion group, the chief surgeon loosened the peri‐patella soft tissue with an electric knife to dissociate the patellofemoral ligament and then flipped the patella (Fig. 3). Other procedures were the same: removing excess peri‐patella synovium and osteophytes, trimming the patella with a narrow oscillating saw, and circumferential electrocautery of the patella.

#### *Prosthesis Implantation*

The meniscus and synovium were completely resected, and part of the fat pad under the tibia was removed. After the ligaments and the posterior joint capsules were released to achieve a primary balance, osteotomy procedures were performed according to the manual. The prosthesis was fixed in place after the flexion and extension gaps were balanced. A posterior‐stabilized prosthesis (Attune, DePuy, Warsaw, Indiana, USA) was implanted. Patellar tracking was checked every time after the implantation of the test mold and after the implantation of the prosthesis. No lateral releases or patella resurfacing were required.

### *Postoperative Management*

Multimodal postoperative pain management and an accelerated physical exercise protocol of our center^16^ were administered to all patients. After admission, patients were given 200 mg celecoxib orally every 12 h for preemptive analgesia until the morning of the operation. Intraoperatively, patients were given 20 mg of ropivacaine diluted with 60 mL of normal saline by topical injection to the joint capsule and collateral ligaments. On postoperative day 1, patients were given parecoxib 40 mg i.v. every 12 h. From postoperative day 2, patients resumed taking oral celecoxib 200 mg every 12 h for for pain relief until postoperative day 21 (when stiches were removed). During the postoperative period, if acute pain occurred (VAS ≥6), 5 mg morphine was immediately given to the affected patients subcutaneously. If moderate pain frequently occurred when patients were exercising (4 ≤ VAS < 6), oxycodone was given to them orally every 12 h. An ice compress was applied to all patients for 12 h to relieve pain and swelling. Exercise protocol: the pressure bandage was removed 18 h postoperatively (PO), at which point patients were asked to start exercises, including SLR, knee flexion, and knee extension 20 times/h for 8 h per day^17^.

### *Outcome Measures*

The primary outcome measures included pain (rVAS and mVAS of anterior knee pain and thigh pain, and opioid consumption) and function (aROM and pROM, the time needed for return to SLR and 90° knee flexion). The secondary outcome measures included operation time, LOS, patella‐related (patellar tilt and baja) and other complications, swelling of the knee, HSS score, and SF‐12 score.

#### *Visual Analogue Scale*

A self‐reported score on the 10‐point visual analog scale was used for the assessment of patients' anterior knee pain and thigh pain. A minimum of 0 indicates no pain; a score of 1–3 indicates mild pain, with the sleep quality of patients not affected; 4–6 indicates moderate pain, with sleep quality affected; 7–10 indicates severe pain, with patients not able to sleep due to pain; the maximum value is 10. The VAS scores were marked by patients themselves on a paper with a graduated line starting at zero (no pain) and ending at 10 (the most painful). Both rVAS and mVAS were collected at eight time points (preoperatively, at 24, 48, and 72 h, 3 weeks, 2 and 6 months, and 1 year PO).

#### *Opioid Consumption*

Opioids are analgesic medications that are commonly used to relieve postoperative pain after TKA. Like the VAS score, opioid consumption was also recorded for evaluation of patients' anterior knee pain and thigh pain after TKA. Two major types of opioids were applied: oral oxycodone and subcutaneous morphine. Opioid consumption was converted to equivalents of oral morphine uniformly for statistical analysis; the conversion factor was 10 mg s.c. injected morphine, which was equal to 30 mg of oral morphine, and 10 mg of oral oxycontin, which was equal to 20 mg of oral morphine ^18^, ^19^. Opioid consumption was recorded at 24, 48, and 72 h PO.

#### *Range of Motion*

The recovery of function after TKA was mainly evaluated using the ROM. The ROM is defined as the difference in the angle between the extreme flexion and extension of the joint. According to different mechanisms, the ROM is divided into aROM and pROM; the former is generated by the active contraction of the muscle and the latter is generated by external forces with no arbitrary movement of the muscle. The knee flexion angle was measured by putting the vertex of the goniometer in the middle of the lower edge of the patella, with the two arms, respectively, pointing to the greater trochanter and the lateral malleolus. Both the aROM and pROM were collected at eight time points (preoperatively, at 24, 48, and 72 h, 3 weeks, 2 and 6 months, and 1 year PO) by an independent observer and every angle was measured three times. Finally, the average was recorded.

#### *Straight‐leg Raise and 90° Knee Flexion*

The time patients took to return to SLR and 90° knee flexion were also recorded to supplement the evaluation of functional recovery after TKA. The return of SLR was defined as elevation of the leg at 45° with the foot dorsiflexed and the knee fully extended without extension lag. The measures applied to evaluate quadriceps function following TKA included knee ROM, return of SLR, and early knee flexion^20^.

#### *Hospital of Special Surgery Score*

The HSS score is a 100‐point scoring system introduced by the Hospital for Special Surgery in 1976. It can accurately evaluate the recovery of the knee, especially the motion of patellofemoral and femorotibial joints before and after knee surgeries. The score is calculated as follows: pain (30 points), function (22 points), range of motion (18 points), muscle strength (10 points), flexion deformity (10 points), and stability (10 points).

#### *Short Form Health Survey Score*

The SF‐12 score is a health‐related quality of life measure. It is a patient‐reported outcome that features questions addressing the multidimensional nature of health. Eight dimensionalities are included in this survey: physical function, role of physiology, role of emotion, mental health, body pain, general health, vitality, and social function. These eight dimensionalities are then divided into two parts: a physical component summary (PCS) and a mental component summary (MCS).

#### *Complications*

To explore the potential inconformity of the rate of patella baja or tilt between the two groups before and after TKA, an independent observer performed standardized weight‐bearing anteroposterior knee radiographs on patients in both groups to evaluate the patella position. Lateral radiographs were performed to measure the patellar height with use of the Insall–Salvati ratio, which was defined as the ratio of the lengths of the patellar tendon to the longest diagonal line of the patella. Patella baja was defined as an Insall–Salvati ratio of <0.8. Swelling of the knee was quantified by the circumference of the superior and inferior border of the patella, which were measured three times, and the average values were recorded.

### *Statistical Analyses*

All data management and statistical analyses were performed using the IBM Statistical Package for Social Sciences version 24.0 software (IBM, Armonk, NY, USA). Categorical variables, such as various postoperative complications, are presented as the number (%) of patients and were analyzed using Pearson's χ^2^‐test or Fisher's exact test. Continuous variables such as ROM, VAS, knee circumference, HSS, SF‐12 scores, opioid consumption, operation time, and LOS are presented as the mean (standard deviation) and were analyzed using independent *t*‐tests or paired *t*‐tests. The level of significance was set at *P* < 0.05.

## Results

### *General Results*

A total of 104 patients who underwent primary unilateral TKA without the intraoperative use of tourniquets and completed a minimum follow‐up of 1 year were finally included in this study. In the eversion group, there were 52 patients, including 30 women and 22 men, and the mean age at the time of surgery was 65.9 ± 7.4 years. In the retraction group, there were 52 patients, including 32 women and 20 men, and the mean age at time of surgery was 67.2 ± 8.1 years. There were no significant differences between the two groups with regard to age, gender, BMI, ASA grade, preoperative aROM and pROM, rVAS and mVAS, knee circumference, HSS score, SF‐12 score, alignment, and IS ratio. Baseline demographic and clinical data are presented in Table 1.

**TABLE 1:** Baseline data

Parameters	Eversion group (*n* = 52)	Retraction group (*n* = 52)	*P‐*value
Age	65.9 ± 7.4	67.2 ± 8.1	0.271
Gender (male/female)	22/30	20/32	0.689
BMI (kg/m^2^)	26.5 ± 3.6	26.8 ± 3.1	0.692
ASA grade (I‐II/III)	40/12	44/8	0.320
aROM (°)	98.3 ± 11.6	97.0 ± 13.3	0.653
pROM (°)	101.2 ± 12.9	100.6 ± 13.1	0.733
rVAS	3.3 ± 0.8	3.2 ± 0.9	0.771
mVAS	5.5 ± 0.7	5.4 ± 1.0	0.875
Knee circumference			
Upper pole of the patella	37.8 ± 1.9	37.9 ± 2.6	0.901
Lower pole of the patella	34.6 ± 1.8	34.1 ± 1.9	0.333
HSS	49.1 ± 8.2	49.4 ± 9.1	0.892
SF‐12			
PCS	31.82 ± 10.35	30.27 ± 10.10	0.743
MCS	42.05 ± 11.14	42.68 ± 11.87	0.901
Alignment (°)	180.7 ± 6.2	178.8 ± 5.7	0.778
IS ratio	1.07 ± 0.13	1.10 ± 0.14	0.539

aROM, active range of motion; ASA, American Society of Anesthesiologists; BMI, body mass index; HSS, Hospital for Special Surgery; IS, Insall‐Salvati; MCS, mental component summary; mVAS, visual analog scale in motion; PCS, physical component summary; pROM, passive range of motion; rVAS, visual analog scale at rest; SF‐12, Short form health survey. The *P*‐value represents the result of one‐way analysis of variance for independent means for continuous variables or the *χ^2^*‐test for independent proportions. The values are presented as the mean and the standard deviation or just the number of different kinds of patients.

### *Visual Analogue Scale*

#### *Visual Analogue Scale* *at Rest*

**Fig. 4:**
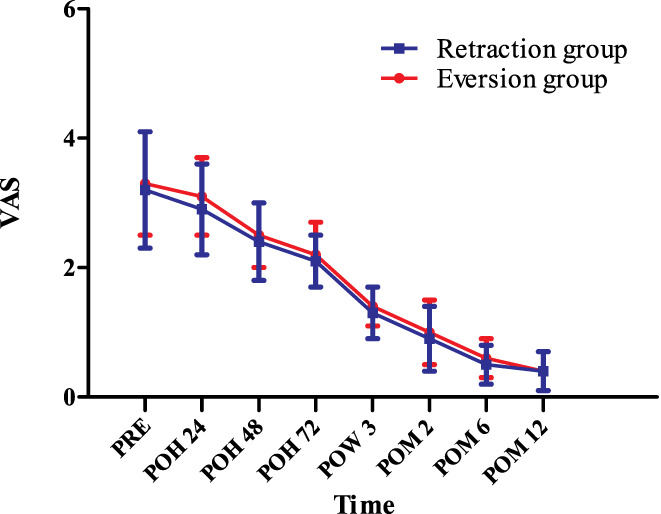
The visual analogue scale score of anterior knee pain at rest (rVAS) at different time points. POH, postoperative hour; POM, postoperative month; POW, postoperative week; PRE, preoperation; VAS, visual analogue scale; * indicates a significant difference (*P* < 0.05). rVAS improved gradually from the preoperative to the predischarge postoperative days and continued to improve during the 1‐year follow‐up. No between‐group differences were found in rVAS at any time point.

The rVAS improved gradually from the preoperative to the predischarge postoperative days and continued to improve during the 1‐year follow‐up. No between‐group differences were found in rVAS at any time point (*P* > 0.05) (Fig. 4), but the VAS of thigh pain in the eversion group was significantly higher at 24, 48, and 72 h PO than those in the retraction group. At 24 h PO, the VAS of thigh pain in the eversion group was 18.2% higher than in the retraction group (2.6 ± 0.8 *vs* 2.2 ± 0.5 [*P* = 0.003]); at 48 h PO, the VAS of thigh pain in the eversion group was 11.1% higher than in the retraction group (2.0 ± 0.5 *vs* 1.8 ± 0.4 [*P* = 0.026]); at 72 h PO, the VAS of thigh pain in the eversion group was 22.2% higher than in the retraction group (1.1 ± 0.4 *vs* 0.9 ± 0.5 [*P* = 0.012]). However, no case of thigh pain was recorded at 3 weeks, 2 or 6 weeks, or 1 year PO (Table 2).

**TABLE 2:** Thigh pain and opioid consumption

Parameters	Eversion group (n = 52)	Retraction group (n = 52)	*P‐*value
VAS (24 h PO)	2.6 ± 0.8	2.2 ± 0.5	0.003^*^
VAS (48 h PO)	2.0 ± 0.5	1.8 ± 0.4	0.026^*^
VAS (72 h PO)	1.1 ± 0.4	0.9 ± 0.5	0.012^*^
VAS (3 weeks PO)	0	0	N.A.
VAS (2 months PO)	0	0	N.A.
VAS (6 months PO)	0	0	N.A.
VAS (1 year PO)	0	0	N.A.
OC (0–24 h PO) (mg)	22.3 ± 7.7	15.1 ± 8.9	<0.001^*^
OC (24–48 h PO) (mg)	27.3 ± 9.3	21.4 ± 10.5	0.003^*^
OC (48–72 h PO) (mg)	23.1 ± 8.2	19.8 ± 7.6	0.036^*^

NA, not applicable; OC, opioid consumption; PO, postoperatively; VAS, visual analog scale.

* Indicates a significant difference. The *P*‐value represents the result of one‐way analysis of variance for independent means for continuous variables. The values are presented as the mean and the standard deviation.

#### *Visual Analogue Scale* *in Motion*

**Fig. 5:**
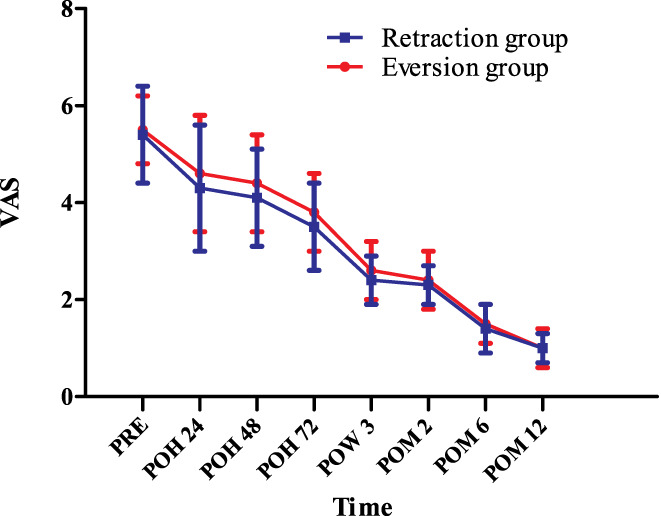
The visual analogue scale score of anterior knee pain in motion (mVAS) at different time points. POH, postoperative hour; POM, postoperative month; POW, postoperative week; PRE, preoperation; VAS, visual analogue scale; * indicates a significant difference (*P* < 0.05). mVAS improved gradually from the preoperative to the predischarge postoperative days and continued to improve during the 1‐year follow up. No between‐group differences were found in mVAS at any time point.

In terms of the mVAS, it also improved gradually from the preoperative to the predischarge postoperative days and continued to improve during the 1‐year follow‐up. No between‐group differences were found with regard to mVAS at any time point (*P* > 0.05) (Fig. 5).

### *Opioid Consumption*

The opioid consumption was significantly higher in the eversion group than in the retraction group on the 3 postoperative days. At 24 h, opioid consumption in the eversion group was 47.7% more than that in the retraction group (22.3 ± 7.7 *vs* 15.1 ± 8.9 mg [*P* < 0.001]); at 48 h, opioid consumption in the eversion group was 27.6% more than that in the retraction group (27.3 ± 9.3 *vs* 21.4 ± 10.5 mg [*P* = 0.003]); at 72 h, opioid consumption in the eversion group was 16.7% more than that in the retraction group (23.1 ± 8.2 *vs* 19.8 ± 7.6 mg [*P* = 0.036]) (Table 2).

### *Range of Motion*

#### *Active Range of Motion*

**Fig. 6:**
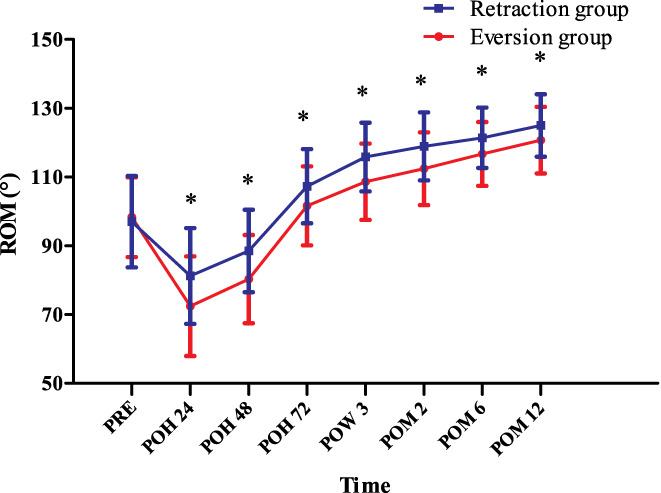
The active range of motion (aROM) at different time points. POH, postoperative hour; POM, postoperative month; POW, postoperative week; PRE, preoperation; ROM, range of motion; * indicates a significant difference (*P* < 0.05). aROM in the two groups increased steadily throughout the predischarge postoperative and follow‐up periods. aROM was significantly higher in the retraction group at all time points postoperatively.

The aROM in the two groups increased steadily throughout the predischarge postoperative and follow‐up periods. Moreover, the aROM was significantly higher in the retraction group at all time points: At 24 h PO, the aROM in the retraction group was 12% better than that in the eversion group (81.2° ± 13.9° *vs* 72.4° ± 14.5° [*P* = 0.002]) and the percentage fluctuated from 3.6% to 12% during the study period. At 48 h PO, the aROM in the retraction group was 10.2% better than that in the eversion group (88.5° ± 12.0° *vs* 80.3° ± 12.8° [*P* = 0.001]); at 72 h PO, the aROM in the retraction group was 5.6% better than that in the eversion group (107.3° ± 10.8° *vs* 101.6° ± 11.5° [*P* = 0.011]); at 3 weeks PO, the aROM in the retraction group was 6.6% better than that in the eversion group (115.8° ± 10.0° *vs* 108.6° ± 11.1° [*P* = 0.001]); at 2 months PO, the aROM in the retraction group was 5.8% better than that in the eversion group (118.9° ± 9.9° *vs* 112.4° ± 10.6° [*P* = 0.002]); at 6 months PO, the aROM in the retraction group was 4.0% better than that in the eversion group (121.4° ± 8.8° *vs* 116.7° ± 9.3° [*P* = 0.009]); at 1 year PO, the aROM in the retraction group was 10.2% better than that in the eversion group (125.0° ± 9.1° *vs* 120.7° ± 9.7° [*P* = 0.022]) (Fig. 6).

#### *Passive Range of Motion*

**Fig. 7:**
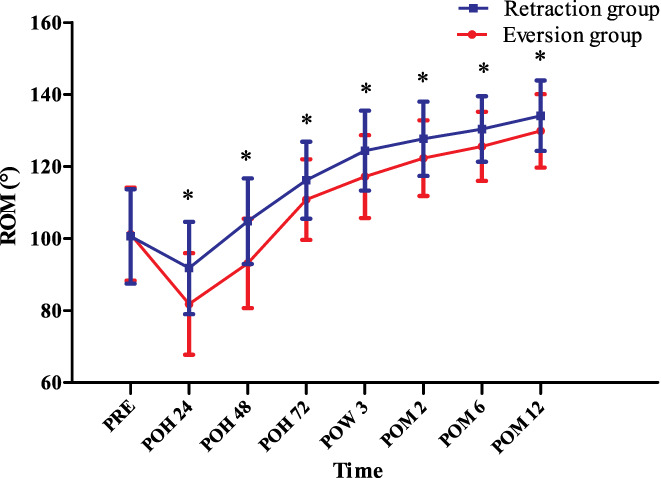
The passive range of motion (pROM) at different time points. POH, postoperative hour; POM, postoperative month; POW, postoperative week; PRE, preoperation; ROM, range of motion; * indicates a significant difference (*P* < 0.05). pROM in the two groups increased steadily throughout the predischarge postoperative and follow‐up periods. Besides, pROM was significantly higher in the retraction group at all time points postoperatively.

In terms of the pROM, in the two groups, the pROM also increased steadily throughout the predischarge postoperative and follow‐up periods. Moreover, the pROM was significantly higher in the retraction group at all time points: patients in the retraction group had approximately 12% more range of motion than that in those in the eversion group at 24 h PO (91.8° ± 12.8° *vs* 81.8° ± 14.1° (*P* < 0.001)) and the percentage fluctuated from 3.2% to 12.6% during the study period. At 48 h PO, the pROM in the retraction group was 12.6% better than that in the eversion group (104.8° ± 11.9° *vs* 93.1° ± 12.4° [*P* < 0.001]); at 72 h PO, the pROM in the retraction group was 4.9% better than that in the eversion group (116.2° ± 10.7° *vs* 110.8° ± 11.2° [*P* = 0.014]); at 3 weeks PO, the pROM in the retraction group was 6.1% better than that in the eversion group (124.4° ± 11.1° *vs* 117.2° ± 11.5° [*P* = 0.002]); at 2 months PO, the pROM in the retraction group was 4.4% better than that in the eversion group (127.7° ± 10.3° *vs* 122.3° ± 10.5° [*P* = 0.009]; at 6 months PO, the pROM in the retraction group was 3.8% better than that in the eversion group (130.4° ± 9.1° *vs* 125.6° ± 9.6° [*P* = 0.01]); at 1 year PO, the pROM in the retraction group was 3.2% better than that in the eversion group (134.1° ± 9.8° *vs* 129.9° ± 10.2° [*P* = 0.035]) (Fig. 7).

### *Straight‐leg Raise and 90° Knee Flexion*

**Fig. 8:**
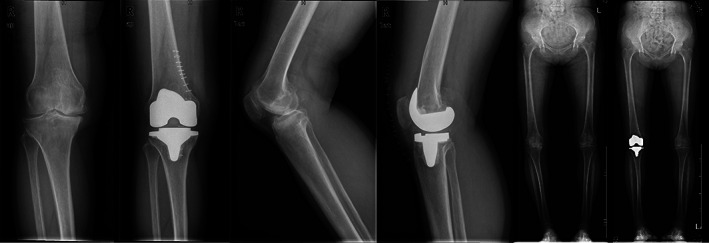
X‐ray images of a patient in the retraction group before and after TKA. The prosthesis is in a good position with no hang of the lateral tibia plateau prosthesis. The line of gravity of the lower limb passes across the medial 1/3 part of the tibia. The patella is in a good position with no patellar baja or patellar tilt.

**Fig. 9:**
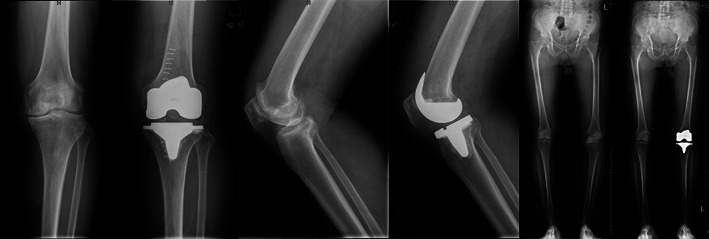
X‐ray images of a patient in the eversion group before and after TKA. The prosthesis is in a good position with no hang of the lateral tibia plateau prosthesis. The line of gravity of the lower limb passes across the medial 1/3 part of the tibia. The patella is in a good position with no patellar baja or patellar tilt.

The time that patients took to return to SLR and 90° knee flexion were both significantly longer in the eversion group than in the retraction group. The time for return of SLR in the eversion group was 15.8% longer than that in the retraction group (2.2 ± 0.8 days *vs* 1.9 ± 0.7 days [*P* = 0.044]); the time for return of 90° knee flexion in the eversion group was 58.3% longer than that in the retraction group (1.9 ± 0.8 days *vs* 1.2 ± 0.7 days [*P* < 0.001]) Figs 8 and 9.

### *Functional Scores*

#### *Hospital for Special Surgery Score*

The HSS scores at 3 weeks and 2 months PO were significantly lower in the eversion group. At 3 weeks PO, the HSS score in the eversion group was 5.6% lower than that in the retraction group (77.4 ± 7.6 *vs* 81.7 ± 7.2 [*P* = 0.004]); at 2 months PO, the HSS score in the eversion group was 4.0% lower than that in the retraction group (85.1 ± 7.2 *vs* 88.5 ± 6.1 [*P* = 0.011]) (Table 3).

**TABLE 3:** Clinical scores

Parameters	Eversion group (*n* = 52)	Retraction group (*n* = 52)	*P‐*value
HSS (3 weeks PO)	77.4 ± 7.6	81.7 ± 7.2	0.004*
HSS (2 months PO)	85.1 ± 7.2	88.5 ± 6.1	0.011*
HSS (6 months PO)	89.2 ± 6.4	90.6 ± 6.0	0.252
HSS (1 year PO)	90.6 ± 5.1	91.3 ± 5.5	0.503
SF‐12 (3 weeks PO)			
PCS	37.1 ± 13.5	39.4 ± 12.5	0.370
MCS	46.1 ± 12.8	47.7 ± 12.2	0.516
SF‐12 (6 months PO)			
PCS	42.5 ± 11.3	43.2 ± 11.7	0.757
MCS	48.8 ± 11.2	49.3 ± 12.1	0.827
SF‐12 (1 year PO)			
PCS	45.1 ± 10.1	45.7 ± 10.6	0.768
MCS	51.5 ± 10.4	52.9 ± 11.5	0.516

HSS, Hospital for Special Surgery; MCS, mental component summary; PCS, physical component summary; PO, postoperatively; SF‐12, Short Form Health Survey.

* Indicates a significant difference. The *P*‐value represents the result of one‐way analysis of variance for independent means for continuous variables. The values are presented as the mean and the standard deviation.

#### *Short Form‐12 Scores*

During the 1‐year follow‐up period, no between‐group differences were found with regard to the SF‐12 score. Both PCS and MCS were comparable when measured 3 weeks, 6 months, and 1 year PO (Table 3).

### *Operation Time and Length of Stay*

A significant difference was found between the eversion group and the retraction group in LOS (5.61 ± 1.92 days *vs* 4.93 ± 1.45 days [*P* = 0.044]). The LOS in the eversion group was 13.8% longer than in the retraction group, but no significant difference was found in operation time (79.23 ± 8.42 min *vs* 77.87 ± 10.52 min [*P* = 0.645]).

### *Complications*

The upper and lower poles of the patella were comparable in the two groups, indicating that eversion being performed or not had nothing to do with postoperative swelling of the knee (Table 4). There was only one case of patella baja in the eversion group (*P* = 1.000). No case of patellar tilt occurred in either group. Five complications occurred in the eversion group, among which one involved minor tearing of the patellar tendon (with the involved width being approximately 20%) and did not need repair; two complications involved incision fat liquefaction and resolved after being treated by more frequent dressing changes; two complications were knee hematomas, which finally healed after conservative treatment with infrared therapy. Four complications occurred in the retraction group. Of these, two patients had incision fat liquefaction, with the patients recovering after receiving more frequent dressing changes. Two were superficial wound infections, but both resolved with antibiotic treatment without progressing to deep infection. The aforementioned complications all occurred in different patients, and no significant difference was found in complication rates between the two groups (Table 5).

**TABLE 4:** Swelling of the knee

Parameters	Eversion group (*n* = 52)	Retraction group (*n* = 52)	*P‐*value
UPP (24 h PO)	39.1 ± 2.0	39.2 ± 2.4	0.818
LPP (24 h PO)	35.7 ± 2.1	35.2 ± 1.9	0.206
UPP (48 h PO)	39.5 ± 1.9	39.4 ± 2.6	0.823
LPP (48 h PO)	36.0 ± 2.0	35.4 ± 1.7	0.102
UPP (72 h PO)	39.6 ± 2.0	39.5 ± 2.5	0.822
LPP (72 h PO)	36.5 ± 1.6	36.1 ± 1.4	0.178

LPP, lower pole of patella; PO, postoperatively; UPP, upper pole of patella. The *P*‐value represents the result of one‐way analysis of variance for independent means for continuous variables. The values are presented as the mean and the standard deviation.

**TABLE 5:** Complications

Parameters	Eversion group (*n* = 52)	Retraction group (*n* = 52)	*P‐*value
Patellar baja	1 (0.02%)	0	1.000
Patellar tilt	0	0	1.000
Patellar tendon tearing	1 (0.02%)	0	1.000
Incision fat liquefaction	2 (0.04%)	2 (0.04%)	1.000
Knee hematoma	2 (0.04%)	0	1.000
Superficial wound infection	0	2 (0.04%)	1.000

The *P*‐value represents the result of the χ^2^‐test or the Fisher test for independent proportions. The values are presented as the number of patients and the percentage.

## Discussion

Without the intraoperative use of a tourniquet, patellar lateral retraction could lead to a superior postoperative outcome compared with conventional patellar eversion during TKA, which was demonstrated in terms of not only pain but also functional outcomes during the predischarge postoperative and 1‐year follow‐up periods by using our primary outcome measures, including rVAS and mVAS of anterior knee pain, VAS of thigh pain, opioid consumption, aROM, pROM, and the time needed for return of SLR and 90° knee flexion, as well as our secondary outcome measures, including LOS and HSS scores.

### *Use of Tourniquet in Total Knee Arthroplasty*

All previous research on handling of the patella in TKA was conducted using an intraoperative tourniquet. However, in recent decades, an increasing number of researchers have found that the use of tourniquets can cause adverse events, including thigh pain, knee swelling, and other ischemia reperfusion injuries, therefore delaying postoperative recovery^13^, ^14^, ^15^. When the tourniquet is used for more than 100 min, the risk of incision complications, DVT, and pulmonary embolism (PE) all significantly increase^15^. Zhou *et al*.^13^ reported that with the use of a tourniquet, latent blood loss after loosening of the tourniquet might flow into the soft tissue, leading to limb swelling and thigh pain, which would, therefore, affect early postoperative rehabilitation. It is worth noting that the adverse events caused by intraoperative tourniquets overlap with the potential complications that follow eversion of the patella, such as thigh pain, poor ROM, delayed return of SLR and 90° flexion, and longer LOS. Therefore, with the confounding factor of the tourniquet, it was difficult to conclude exactly what caused the aforementioned adverse events and to identify the difference in outcomes for different techniques for handling the patella. For the first time, we found in this study that without the intraoperative use of a tourniquet, patellar lateral retraction is superior to eversion in terms of the clinical outcomes after TKA.

### *Postoperative Anterior Knee Pain*

In terms of postoperative anterior knee pain, we found no significant difference between the eversion and retraction groups, as reported in other studies. Jenkins *et al*.^8^ found no difference in the postoperative knee pain VAS score at 48 h PO between the retraction and eversion groups. Reid *et al*.^9^ reported that although the VAS score decreased from 3 months PO to 1 year PO, and there was a trend toward a lower VAS score in the retraction group, the difference did not reach significance. Some others reported significantly more severe anterior knee pain in the eversion group. For example, Majima *et al*.^5^ found that the non‐eversion group showed a lower VAS score than the eversion group until 4 weeks PO. The reason why previous studies drew contradictory conclusions might be due to the inconsistent use of tourniquets, which made patients in both eversion and retraction groups develop different levels of lower‐limb ischemia reperfusion injury, which might affect the evaluation of anterior knee pain. Another reason might be that anterior knee pain was measured by the VAS, which is a subjective scale scored by patients themselves. Some patients simply misconstrued thigh pain and anterior knee pain and provided an inaccurate VAS value for anterior knee pain.

### *Postoperative Thigh Pain*

In this study, we not only avoided the potential effect of tourniquets but also emphasized the accurate evaluation of anterior knee pain distinguished from thigh pain. We found that patients in the eversion group experienced significantly more severe thigh pain, which could explain the comparable anterior knee pain between the two groups but the significantly greater opioid consumption in the eversion group. It was thigh pain that increased the opioid consumption of patients in the eversion group. In addition, the results of our study showed that although thigh pain in the eversion group was significantly more severe at 24, 48, and 72 h PO, no case of thigh pain occurred during the subsequent 1‐year follow‐up period (3 weeks, 2 and 6 months, and 1 year PO) in either group, which suggested that thigh pain was a type of early pain caused by intraoperative handling of the patella. The reason why patellar eversion caused more severe thigh pain might be that when performing patellar eversion intraoperatively, an extra pulling force on the quadriceps femoris was generated, which increased torsion stress to the quadriceps femoris and, therefore, increased the incidence and degree of early postoperative thigh pain in some patients. However, intraoperative traction did not cause any real damage to the quadriceps femoris, which is why thigh pain only existed temporarily (for a few days PO). Therefore, we concluded that eversion or lateral retraction of the patella in tourniquet‐free TKA had no influence on anterior knee pain but that patellar eversion increased early thigh pain and opioid consumption in patients.

### *Range of Motion*

In this study, we found that after tourniquet‐free TKA, the ROM (both aROM and pROM) was significantly worse in the eversion group at all time points. There are two possible reasons. First, as mentioned above, patients who received patellar eversion were recorded as having significantly more severe thigh pain for a few days PO. The pain might have affected the patients in relation to both active and passive motion of the knee because once the knee flexed, whether actively or passively, the quadriceps would tense, causing pain. Second, the different ROM might also be attributed to the absence of excessive traction on the extensor mechanism because traction on the extensor mechanism in combination with excessive hyperflexion of the knee might lead to invisible damage to the extensor mechanism, which might, therefore, cause subsequent fibrosis of the quadriceps and patellar tendon and limit the motion of the knee^3^. The worse ROM existed until the last follow‐up at 1 year PO, suggesting that the adverse effect of patellar eversion on ROM might be permanent, which calls for further follow‐up.

As previous studies reported, the measures applied to evaluate quadriceps function following TKA included knee ROM, return of SLR and early knee flexion, and LOS^20^. In terms of SLR, all the published papers that took SLR into account concluded that patellar lateral retraction could provide a better return of SLR than patellar eversion^3^, ^4^, ^8^, ^20^, ^21^, which was in accordance with our results. For early knee flexion, there is some research in favor of better flexion after lateral retraction^22^, similar to our results. Regarding LOS, Jenkins *et al*.^8^ reported a significantly longer LOS in an eversion group (4.8 ± 2.6 *vs* 4.0 ± 1.4 [*P* = 0.03]), which was not in accord with the results of Reid *et al*.^9^ but was comparable with ours. Therefore, we concluded that patellar lateral retraction was better for quadriceps function recovery. In contrast, Umrani *et al*.^23^ directly measured the quadriceps force and power with a dynamometer and recorded a comparable result in eversion and retraction groups. However, they also applied tourniquets in all TKA. Even if they used the dynamometer to provide relatively objective data, they could not exclude ischemia reperfusion injury caused by the intraoperative use of a tourniquet, which might affect the evaluation of quadriceps function as a confounding factor.

### *Complications*

In the eversion group, one patient exhibited patella baja and another patient experienced partial tearing (accounting for less than 20%) of the patellar tendon, while no case of patella baja or patellar tendon injury occurred in the retraction group. Although the difference did not reach clinical significance, there was a trend of patella‐related complications in patients undergoing TKA with intraoperative patellar eversion.

### *Limitations*

A few limitations should be noted. First, the 1‐year follow‐up time of our study may not be sufficient for certain outcomes, such as aROM and pROM, which need longer follow‐ups to confirm whether the difference in the ROM between the two groups still exists after a longer time period. However, our first postoperative outcome measures and data collection started at 24 h PO and then continued at 24‐h intervals, which is the earliest in the series studies and has the continuity of the predischarge postoperative observation of clinical outcomes. Second, we recorded the ROM, return of SLR and early knee flexion, and LOS to reflect quadriceps function recovery. Further studies could use a dynamometer to provide objective and reproducible data regarding quadriceps force and power when investigating the recovery of quadriceps function after tourniquet‐free TKA.

### *Conclusion*

On the basis of our results, we found significantly better ROM, earlier return of SLR, and shorter LOS in the patellar lateral retraction group. Although postoperative anterior knee pain was comparable in the two groups, patients in the patellar eversion group experienced significantly more severe thigh pain and consumed more opioids. In addition, patellar eversion may have resulted in a slightly higher rate of complications with regard to patella baja and tendon injury, even if the difference in rates did not reach clinical significance. Therefore, we finally recommend lateral retraction rather than eversion for optimal handling of the patella in TKA.
